# Adrenal crisis mainly manifested as recurrent syncope secondary to tislelizumab: a case report and literature review

**DOI:** 10.3389/fimmu.2023.1295310

**Published:** 2024-01-16

**Authors:** Haishan Wei, Anju Zuo, Jiying Chen, Chunyan Zheng, Tingting Li, Haiyan Yu, Yuan Guo

**Affiliations:** Department of General Practice, Qilu Hospital of Shandong University, Jinan, China

**Keywords:** tislelizumab, immune-related adverse events (irAEs), adrenal crisis, immune checkpoint inhibitors (ICIs), recurrent syncope

## Abstract

As an immune checkpoint inhibitor (ICI), tislelizumab is an anti-programmed cell death protein 1 (PD-1) drug. With the extensive application of ICIs, there is an ever-increasing proportion of immune-related adverse events (irAEs) in clinical settings, some of which may even be life-threatening. Herein, we present a patient with tislelizumab-induced adrenal crisis. The main clinical manifestation was recurrent syncope accompanied by high-grade fever. Timely identification and hormone replacement therapy helped the patient overcome the crisis well. Finally, the patient discontinued tislelizumab and switched to antibody–drug conjugate (ADC) therapy. We report this case to improve our understanding of this situation, identify this kind of disease, and prevent adrenal crisis in time. Eventually, limiting toxicities reduces the interruption of immunotherapy. Since irAEs are multisystem damage with more non-specific symptoms, except for oncologists, general practitioners who endorse the need for taking a holistic approach to the patient should play a vital role in the management of cancer treatment.

## Introduction

Nowadays, the treatment of multiple malignancies has been revolutionized by immune checkpoint inhibitors (ICIs), which prolong patients’ long-term survival and produce durable remissions. ICIs are monoclonal antibodies that target two key signaling pathways related to T-cell activation and exhaustion by binding and inhibiting cytotoxic T lymphocyte antigen (CTLA)-4 or programmed death (PD)-1 and its ligand PD-L1 (1, 2). However, ICIs may also demolish the maintenance of immunological tolerance to self-antigens (3), leading to immune-related adverse events (irAEs) in different organ systems, especially autoimmune-like manifestations targeting endocrine glands (4). These toxic effects are a major cause of onset, often leading to treatment discontinuation, and can have debilitating long-term consequences (1). Endocrine dysfunction is one of the most commonly reported irAEs in ICI clinical trials, including hypothyroidism, hyperthyroidism, hypophysitis, primary adrenal hypofunction (PAI), and type 1 diabetes (5).

Little is known about severe adrenal insufficiency (AI) related to ICIs, with an incidence rate of ≤1% (6–9). AI usually manifests as grade 1–2 irAEs, while adrenal crisis (AC) manifesting as grade 3–4 irAEs is rare. The presentation of AI is usually non-specific. The main clinical symptoms include fatigue, anorexia, and nausea, which may be misdiagnosed as complications of a malignant tumor. When AI is not recognized, misdiagnosis or delayed diagnosis may lead to life-threatening AC (10, 11). A history of previous AC is a susceptible factor for patients with AI to experience AC again (10). Severe symptoms of adrenal crisis may lead to a decline in confidence and discontinuation of immunotherapy. Therefore, it is of great clinical significance to identify and treat AI in time.

This case report describes a middle-aged man with non-invasive urothelial carcinoma who manifested AC characterized by recurrent syncopal episodes after treatment with a PD-1 inhibitor, tislelizumab. Syncope under the category of undifferentiated symptom diseases necessitates a significant investment of time, finances, and effort to pinpoint the precise etiology (12). We present this case to underscore the importance of pre-medication education and regular post-usage monitoring of relevant diagnostic parameters. Elevating the awareness of healthcare practitioners regarding adverse drug reactions contributes to minimizing the progression of such reactions, ultimately reducing the temporal and financial costs incurred by patients.

## Case description

A 58-year-old male patient was admitted to our department due to recurring syncopal episodes for more than 3 months. He was also suffering from high fever, confusion, fatigue, anorexia, nausea, and vomiting. The patient’s family once monitored his blood pressure after syncope with a systolic blood pressure of 50–60 mmHg and a blood glucose level of 4.6 mmol/L. In addition to physical symptoms, the patient was under great mental stress at the time of admission.

**Figure 1:**
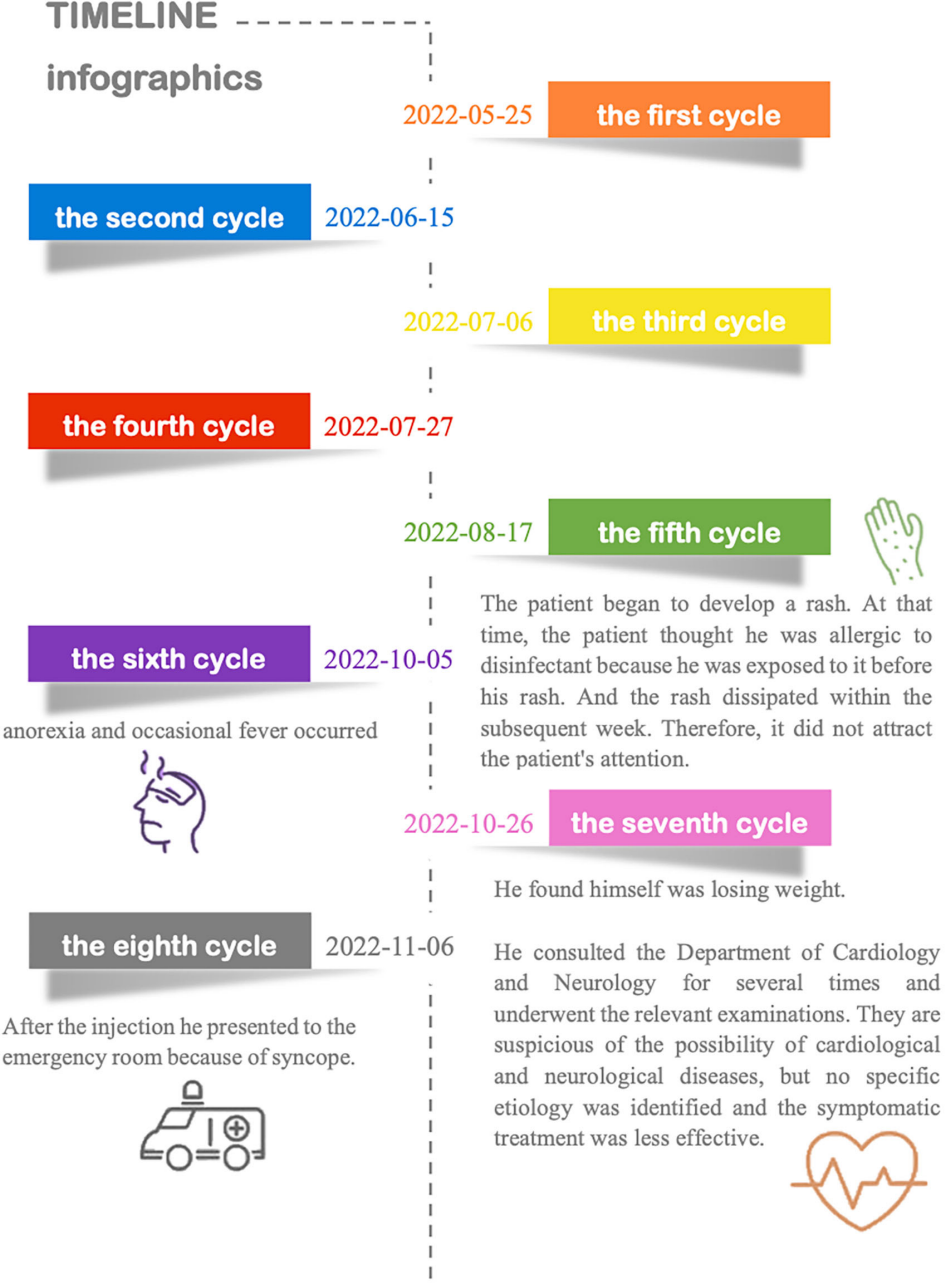
Timeline of symptoms.

Three years ago, he was diagnosed with urothelial carcinoma and underwent minimally invasive surgery. The tumor recurred 6 months after resection, and on May 20, 2022, he underwent complete transurethral resection of bladder tumor (TURBT). Histological diagnosis was low-grade non-invasive papillary urothelial carcinoma. Cancer tumor staging showed no metastasis or local invasion, and the last contrast-enhanced multislice computed tomography (CT) was normal. The patient received treatment with tislelizumab (approximately eight cycles). The first four cycles of immunotherapy were from May 25, 2022, to July 27, 2022, with the infusion of tislelizumab (200 mg, injection d1 3 weeks) without specific discomfort. Further details and information are presented in **Figure 1**. He has a smoking history for more than 30 years. Previous endocrine disorders were unclear.

On admission, his body temperature (BT) was 35.5°C, and his blood pressure (BP) was 117/77 mmHg. His physical examination revealed increased breath sounds and a positive Murphy’s sign. Combined with the results of previous laboratory examinations and clinical manifestations, we first consider the possibility of Adams–Stokes syndrome attack, viral myocarditis, transient ischemic attack (TIA), pulmonary embolism, sepsis, vasovagal syncope, insulinoma, PD-1-related adverse reactions, and so on.

Laboratory data revealed a high level of high-sensitivity C-reactive protein. We further performed contrast-enhanced multislice CT, which showed interstitial infiltrates and exaggerated lung markings. The level of serum sodium was normal. Other abnormal data are shown in **Table 1**. Diurnal rhythm changes of serum adrenocorticotropic hormone (ACTH) and cortisol suggested extremely low cortisol and ACTH and inconsistent diurnal rhythm (**Table 2**), considering that the patient had hypoadrenalism. In view of the low level of ACTH and no abnormalities seen on adrenal CT, the diagnosis of central hypoadrenalism was confirmed. Given the history of immunotherapy, we considered the possibility of immune-related hypophysitis (irH). Consequently, we comprehensively evaluated the endocrine system of patients, as irH could involve the hypothalamic pituitary thyroid axis and gonadal axis in addition to the cumulative hypothalamic–pituitary–adrenal (HPA) axis. Thyroid function tests revealed that although thyroglobulin was slightly elevated, free triiodothyronine (FT3), free thyroxine (FT4), and thyroid-stimulating hormone (TSH) were normal, and thyroid peroxidase and thyrotropin receptor antibodies were negative, suggesting normal pituitary thyroid function. The results of the sex hormone test showed that luteinizing hormone and progesterone were mildly elevated, and the remaining indexes were within normal limits, suggesting normal pituitary–gonadal function. To this point, the patient’s etiology could be clarified, as irH triggered isolated adrenocorticotropic hormone deficiency (IAD). The common clinical manifestations of IAD were fatigue, nausea and vomiting, weight loss, hypoglycemia, hyponatremia, and refractory hypotension. The patient’s symptoms were highly consistent with IAD.

**Table 1:** Laboratory measurements.

Parameter	Value	Normal range
High-sensitivity C-reactive protein (mg/L)	42.98	0–10
Blood glucose (mmol/L)	4.01	3.90–6.10
Sodium (mmol/L)	142	137–147
Potassium (mmol/L)	3.69	3.50–5.30
Glomerular filtration rate (ml/min)	105.35	
Progesterone (nmol/L)	0.590	<0.474
Luteinizing hormone (mIU/ml)	9.04	1.70–8.60
Thyroglobulin (ng/ml)	95.06	1.40–78.0
Ferritin (ng/ml)	818.00	13–400
Neuron-specific enolase (ng/ml)	16.50	0.0–16.3

**Table 2:** Diurnal rhythm changes of serum ACTH and cortisol.

	8 a.m.	4 p.m.	0 a.m.
Cortisol (μg/dl)	0.16	0.20	0.15
ACTH (pg/ml)	4.47	2.37	2.16

ACTH, adrenocorticotropic hormone.

**Figure 2:**
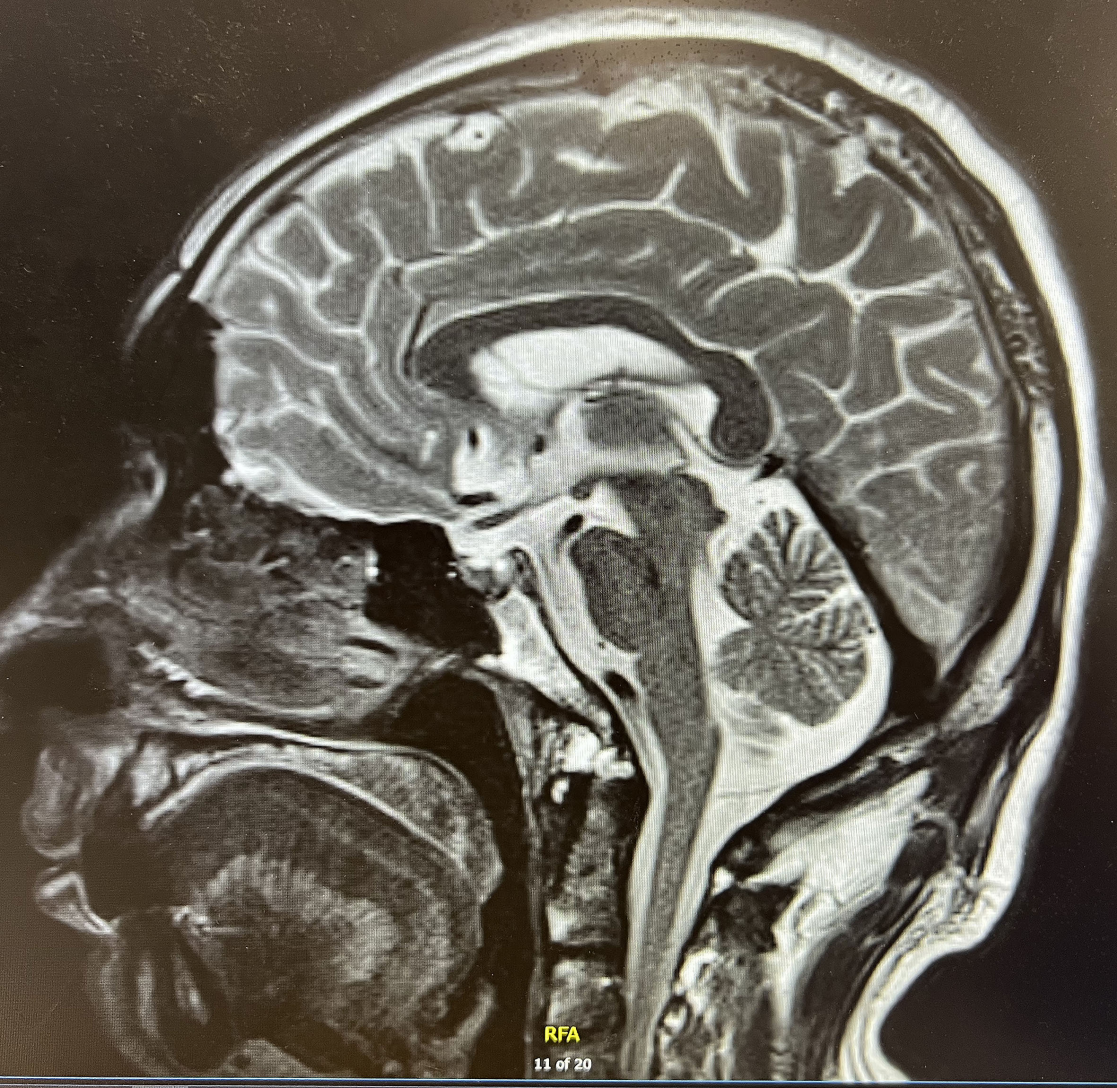
Post-contrast T2-weighted MR image of the pituitary gland.

Finally, we performed pituitary MR imaging (**Figure 2**), which revealed a normal pituitary gland. The patient’s family revealed that the patient experienced absolute low blood pressure (<100 mmHg) and hyperthermia with confusion during the syncopal episode; thus, adrenal crisis was the most reasonable diagnosis. After administration of hydrocortisone sodium succinate (0.15 g iv drip bid) and continuous fluid resuscitation, the patient’s condition gradually improved. After discharge, he continued to be given prednisone 10 mg qd (8a) and 5 mg qd (5p) orally. After 3 months’ follow-up, the patient did not have syncope again, and the symptoms of fever, fatigue, anorexia, nausea, and vomiting improved significantly. In addition, there was no recurrence of adrenal crisis or other immune-related adverse symptoms during the follow-up period.

## Discussion

Here, we introduced a case of adrenal crisis after treatment with PD-1 (tislelizumab), which was a 3–4 grade irAE related to PD-1. Several cases of immunotherapy-induced adrenal crisis have been reported, most of which manifested as high-grade fever, persistent hyponatremia, or acute abdomen, while recurrent syncope is rare, and non-specific symptoms made the diagnosis of diseases difficult. Due to enormous psychological pressure, despite its immense clinical benefits, the patient stopped treatment. Clinical physicians should develop an awareness of irAEs in order to identify the events timely and reduce incidences of discontinuation of ICIs.

According to the American Society of Clinical Oncology (ASCO) Guideline (13), a routine endocrine examination should be taken to evaluate the endocrine gland or organ. In this case, we confirmed the diagnosis of central hypoadrenocorticism through endocrine examination. We then traced the patients’ medical history to figure out the potential cause. The patient had no previous history of taking, inhaling, or injecting steroids and no history of opioid use. In addition to being treated with PD-1 for eight cycles, there were no other relevant reasons and incentives. Therefore, we considered whether there were PD-1-related adverse drug reactions. Among them, irH has attracted our attention, which is defined by the occurrence, in patients treated with ICIs, of functional defect of one or more pituitary axes with or without slight pituitary MRI abnormalities (14). The exact pathogenesis of irH is still unknown. CTLA-4 and PD-1/PDL-1-related hypophysitis are currently known to have different clinical features, which may suggest different underlying mechanisms. CTLA-4-related hypophysitis manifests as frequent impaired TSH and luteinizing hormone/follicle-stimulating hormone (LH/FSH) secretion accompanied by impaired ACTH secretion (15) and a greater propensity for type II hypersensitivity reactions associated with off-target effects of CTLA-4 in the pituitary (16). In contrast, PD-1-associated hypophysitis is less frequent (17), and most patients have a specific impairment of ACTH only, presenting as IAD (18). The pituitary gland of autopsy cases showed evidence of type IV hypersensitivity by cytotoxic T lymphocytes (16). Different clinical presentations are presented depending on the specific target gland axis of injury.

Due to the lack of specific clinical manifestations and accurate onset time, the diagnosis of irH is difficult. At present, the diagnosis is mainly based on biochemical and imaging examinations. Specific immune biomarkers for its diagnosis are not currently available, with the most common biochemical evidence being a deficiency of pituitary hormones. Imaging can rely on pituitary MRI to provide diagnostic evidence: pituitary enlargement, stalk thickening, and enhancement with allogeneic or heterologous contrast media are present on MRI in 77% of patients with IRH, whereas 23%–33% of patients do not show abnormalities on MRI (16). Multiple studies have suggested that hypophysitis induced by PD-1/PD-L1 inhibitors may lack the typical pituitary enlargement compared to CTLA-4 inhibitors (19–21). Therefore, imaging studies showing a normal appearance of the pituitary gland do not rule out hypophysitis (22). In addition, the diagnosis of hypophysitis may lag a few weeks after imaging shows pituitary enlargement (23).

According to the 2022 National Comprehensive Cancer Network (NCCN) guidelines for irH, MRI should be performed if the patient has symptoms during treatment (24). A recent study suggested that brain MRI after receiving ICI therapy should be compared with previous results to monitor changes in pituitary size, which may foreshadow that impending anterior pituitary hormone dysfunction is about to occur (22). An enlarged pituitary gland, as indicated by imaging studies, is important to exclude metastatic disease in addition to suspecting hypophysitis (23). Several studies showed that ICI-related central adrenocortical dysfunction appears to be permanent (22, 23, 25–27). However, most of these reports were central hypoadrenocorticism caused by another immune checkpoint inhibitor CTLA-4 drug. So far, there is a lack of histocytological evidence to prove whether pituitary adrenal axis function could be restored (28, 29). To sum up, hormone replacement therapy should not be delayed by waiting for a pituitary gland MRI when an endocrine examination prompts central hypoadrenocorticism (30).

The initial clinical manifestations of IAD lack specificity, which delays diagnosis and eventually progresses to adrenal crisis, threatening the patient’s life. The main clinical manifestations of adrenal crisis are severe hypotension or hypovolemic shock, acute abdomen symptoms, vomiting, hyperthermia or hypothermia, and hypoglycemia. Among them, hypotension is a core symptom in the diagnosis of adrenal crisis. However, seemingly normal blood pressure could not rule out a crisis. According to the adverse event evaluation criteria (Common Terminology Criteria for Adverse Events (CTCAE)), adrenocortical insufficiency is usually grade 1–2 irAEs, while grade 3–4 irAEs, especially adrenal crisis, are rarely reported. In a large meta-analysis study containing 160 clinical trials and 40,432 patients, Jingli et al. found that among patients using ICIs, the incidence of all-grade and severe-grade hypoadrenalism was 2.43% and 0.15%, respectively (6). Our case had grade 3–4 irAEs induced by tislelizumab who presented with adrenal crisis. The symptoms of our patient were unremarkable and could be easily overlooked if an irAE had not been suspected. Although adrenal crisis is rare, it is a life-threatening side effect of ICIs that requires immediate recognition and treatment with intravenous glucocorticoids. Therefore, a deep understanding of irAEs as well as adrenal crisis, early diagnosis, and treatment is significantly important. When an immunotherapy-related adrenal crisis occurs, an initial intravenous or intramuscular bolus of 100 mg hydrocortisone in addition to supportive fluid therapy is required, as well as a continuous intravenous infusion of 200 mg hydrocortisone q24h (daily) or an intravenous or intramuscular bolus of 50 mg hydrocortisone q6h (or 50 mg four times daily) (10).

The recommended duration is 24–48 h until the patient can take oral hydrocortisone (11). Glucocorticoid replacement therapy should be the primary treatment when the patient’s condition is stable. Our patient had no history of underlying endocrine diseases such as diabetes, so there were no specific restrictions on the dose of cortisol to be administered. In patients with diabetes, choosing the appropriate cortisol dose that in turn maximizes benefits and reduces associated side effects is a challenge. Considering that high-dose cortisol may aggravate the underlying disease or lead to new disease (31), the potential benefit of high-dose glucocorticoid treatment should be balanced against efficacy loss due to anticancer immunotherapy. Although this issue remains controversial (27), the dose of cortisol should be reduced appropriately. For adults, the oral maintenance dose of hydrocortisone needs to be 15–25 mg per day (32). The hydrocortisone dose should then be gradually reduced according to the patient’s clinical manifestations, with close monitoring of blood pressure and recurrence of clinical symptoms (33).

For patients who develop endocrine diseases that can be controlled using hormone replacement therapy, there is no need to discontinue ICIs despite grade 3–4 irAEs (34, 35). Theoretically, during the treatment period of ICIs, at the same time as the immune system’s reduced tolerance to triggering irAEs, its ability to recognize and kill cancer cells is enhanced, so the occurrence of irAEs may be a positive predictor of treatment response (22). Our patient underwent imaging examinations that showed no metastasis and recurrence of the tumor, indicating that this patient achieved a complete response to the treatment with tislelizumab. Meanwhile, several studies have shown a positive correlation between the development of irAEs as a result of ICI therapy and improved tumor response and survival. However, for grade 3–4 irAEs, life-threatening side effects require urgent hospitalization for corresponding symptomatic supportive care. After adverse reaction disappearance, the restoration of ICIs requires consideration of many situations, such as previous tumor reactions, treatment duration, toxicity type and severity, toxicity resolution time, availability of alternative therapies, and patient’s condition (13). Multiple studies have confirmed a significantly increased incidence of irAEs with the combination of ICIs, and it is not recommended to switch to a new ICI (27, 36–38). After a consult with an oncologist on this patient’s condition, the oncologist recommended an antibody–drug conjugate (ADC) therapy. At our later follow-up visit, the patient decided to discontinue ICI therapy and switch to ADC to continue the anti-tumor treatment.

ADCs are composed of monoclonal antibodies, cytotoxic payloads, and linkers (39, 40). The efficacy and toxicities of an ADC as a cytotoxic therapy are contingent upon the critical contributions of each component (40, 41). Although high specificity and low toxicity are expected for this novel compound, unpredictable toxicity still exists and demands prompt consideration (40, 42). In the subsequent follow-up, our patient still exhibited adrenal insufficiency, but common side effects attributable to ADC drugs were not observed, such as thrombocytopenia, liver or ocular toxicity, and peripheral neuropathy (42). At present, a subset of clinical trials is underway for combined regimens involving ADC drugs and immune checkpoint inhibitors (43, 44). Additionally, it underscores the importance of clinicians exercising caution with respect to the drug toxicities induced by the combined regimen.

In addition, a growing number of clinical cases prove that endocrine diseases such as late-onset AC still occur after the termination of ICI therapy (45, 46), which also proves that the anti-tumor effects of ICIs can be long-term *in vivo* and expressed (46, 47). Therefore, it is recommended to be always alert to the possibility of irAEs even after the discontinuation of ICIs. Current medical examination methods cannot distinguish between immunological- and non-immunological-related causes and specific immunological biomarkers deprivation, making it difficult for clinicians to detect irAEs (48). Because of their specificity of presentation, atypical timing, and clinical coexisting with other diseases, irAEs may be more difficult to diagnose and identify (48–50). Especially for immune checkpoint inhibitors, risk factors predicting these events have yet to be determined. It is a challenge to predict who will develop severe or permanent toxicity (1). Before giving treatment to patients with PD-1/PD-L1 inhibitors, it is necessary to inquire about the history of endocrine diseases and autoimmune diseases in detail, conduct reasonable baseline screening, regularly monitor changes in endocrine indicators, increase vigilance against possible related symptoms and signs, and detect and promptly handle irAEs as soon as possible (22, 51). Once the dose of hormones and the types of anti-tumor drugs are determined, it is necessary to further provide patients with knowledge of common adverse reactions to ICI and conduct regular follow-up visits. We believe that self-education and management of such patients play an important role in the progress of the disease (52). Timely identification limits toxicities while maximizing anti-tumor efficacy to reduce the interruption of immunotherapy.

When reviewing the patient’s history, it was found that the patient had skin manifestations of irAEs 5 months before admission. However, these failed to capture the attention of both the patient and clinicians. Despite that the most common organ system was involved (1, 5), the initial warning symptom was ignored, resulting in consequential outcomes. According to the American Society of Clinical Oncology Clinical Practice Guideline, timely and latest education about immunotherapies should be provided throughout treatment and survivorship (34). However, our patient was only informed that this drug has durable therapeutic effects before treatment, accompanied by a spectrum of side effects affecting different organ systems. The detailed elaboration on these side effects was withheld due to multifarious factors. This case confirms significant deficiencies in our current approaches to education and management. This also poses a question: following a comprehensive explanation of the toxicities associated with ICIs and ADCs, which pharmaceutical approach do patients exhibit a greater inclination to for anti-tumor therapy? However, considering the preexistence of significant side effects, further inquiry at this juncture might compromise the objectivity of the responses from the patients.

With the increase in clinical practice of tumor immunotherapy, the occurrence of immune-related adverse reactions will constantly increase. Specialists of different departments may receive referrals for patients suffering from specific symptoms of adverse events in their field of expertise. However, as irAEs are multisystem damage with more non-specific symptoms, except for specialists, general practitioners should play a greater role in the management of cancer treatment. Identifying and characterizing irAEs is a cornerstone in ascertaining the impact of cancer treatment on patients and healthcare professionals (53). Cancer survivors are often troubled by the long-term consequences of cancer and its treatment (54). Because primary care is an integrated and accessible healthcare service, most patients consult general practitioners initially once they have symptoms. Lower-grade irAEs can be identified and controlled so as to divert medical resource pressure and financial pressure away from tertiary healthcare toward primary healthcare. As gatekeepers to further services, general practitioners should play a greater role in improving the quality of care for cancer survivors.

## Data availability statement

The original contributions presented in the study are included in the article/supplementary material. Further inquiries can be directed to the corresponding author.

## Ethics statement

Written informed consent was obtained from the individual(s) for the publication of any potentially identifiable images or data included in this article.

## Author contributions

HW: Writing – original draft. AZ: Writing – review & editing. JC: Writing – review & editing. CZ: Writing – review & editing. TL: Writing – review & editing. HY: Resources, Writing – review & editing. YG: Writing – review & editing.
